# The Relationship Between Vitamin D Status and the Clinical Severity of COVID-19 Infection: A Retrospective Single-Center Analysis

**DOI:** 10.7759/cureus.22385

**Published:** 2022-02-19

**Authors:** Christiana Zidrou, Angelo V Vasiliadis, Maria Tsatlidou, Maria Sentona, Stavros Vogiatzis, Anastasios Beletsiotis

**Affiliations:** 1 2nd Orthopaedic Department, General Hospital of Thessaloniki “Papageorgiou”, Thessaloniki, GRC; 2 Pulmonology Department, General Hospital of Thessaloniki "Papageorgiou", Thessaloniki, GRC; 3 2nd Orthopaedic Department, General Hospital of Thessaloniki "Papageorgiou", Thessaloniki, GRC

**Keywords:** hospital discharge, inflammatory markers, mortality, covid-19, vitamin d

## Abstract

Background and objective

Some studies have suggested a potential protective role of vitamin D in coronavirus disease 2019 (COVID-19) patients, and this has led to a debate on the topic in the medical community. However, the reported data on the number of hospitalized patients who were vitamin D-deficient is not convincing. In light of this, the aim of the present study was to explore if vitamin D deficiency is correlated with severity and mortality rates of COVID-19 infection in hospitalized COVID-19 patients at a tertiary care hospital in Greece.

Methods

We conducted a single-center retrospective study involving 71 patients hospitalized with COVID-19 from August to October 2020. Serum 25-hydroxyvitamin D (25(OH)D) level was assessed in all patients within 48 hours of hospital admission. Serum 25(OH)D level ≤20 ng/ml was defined as a deficiency, while that >20 ng/ml as repletion. The primary outcomes of the infection were classified as partial/complete recovery and mortality during hospitalization. The secondary outcomes were blood markers of inflammation and thrombosis.

Results

Among the 71 COVID-19-positive patients [mean age: 63 years, range: 20-97; male (n=47; 66.2%): female (n=24; 33.8%)] who were enrolled in the study, 46 (64.8%) patients had 25(OH)D levels ≤20 ng/ml and 25 (35.2%) had a level >20 ng/ml. According to the patients' medical history, 55 patients (77.5%) had comorbidities. It appears that vitamin D deficiency (<20 ng/ml) significantly correlated with elevated biochemical markers such as procalcitonin and troponin (p<0.001). Moreover, male gender, advanced age (>60 years), and comorbidities were positively associated with more severe COVID-19 infection (elevated inflammation markers, radiographic findings on X-rays, and increased length of hospital stay).

Conclusion

These preliminary findings show that vitamin D status among the patients was not related to the severity of COVID-19 infection.

## Introduction

The symptoms of patients infected with coronavirus disease 2019 (COVID-19) range from being asymptomatic to mild-moderate symptoms including fever, anosmia, dysgeusia, runny nose, sore throat, and cough, as well as severe respiratory illness presenting as shortness of breath with consequent multiple organ failure or death [1]. Advanced age, the presence of comorbidities, and male gender have been shown to be correlated with severe COVID-19 infection [1-3].

The association between vitamin D status and the risk of serious COVID-19, the increase of inflammatory markers, the frequency of complications, as well as mortality remain controversial [4-5]. Research from the University of Chicago reports an increased number of positive COVID-19 test results among patients with vitamin D deficiency [4]. On the other hand, a prospective study by Pizzini et al. has argued that there was no relationship between low 25(OH)D levels and the severity of infection, abnormalities in imaging examinations, and elevated inflammation indicators [5]. Two studies from the UK [6] and Iran [7] have revealed that lower 25(OH)D levels were strongly associated with a greater extent of lung involvement, admission to ICUs, and increased indicators of infection among COVID-19 patients.

The existing literature provides some indications regarding the association between vitamin D deficiency and the severity of COVID-19 infection [8]. Moreover, Daneshkhah et al. have suggested the possible role of vitamin D in decreasing cytokine storm and unregulated infection in COVID-19 patients [9]. If these correlations are confirmed, they may point to a possible role of vitamin D supplements in terms of prevention, prognosis, and treatment of COVID-19 infection.

Furthermore, Martineau et al. have proposed that taking vitamin D supplements has a protective role against acute respiratory inflammations, especially in patients whose vitamin D status is very low (less than 10 ng/ml) [10]. This study has led to a lively debate on the possible consequences of vitamin D deficiency on the clinical outcome and the mortality from COVID-19 infection and on the administration of vitamin D supplements as a potential therapeutic strategy in COVID-19 patients [11-13]. In this study, we aimed to investigate if vitamin D deficiency is associated with the severity of COVID-19 infection and mortality in hospitalized COVID-19 patients at a tertiary referral hospital.

## Materials and methods

Study design and population

This cross-sectional study was conducted at a tertiary care hospital during the three-month period from August to October 2020 after obtaining approval from the Hospital Ethics Committee (345/April 2021). The exclusion criteria were as follows: (i) patients hospitalized with COVID-19 for less than 48 hours, (ii) patients aged ≤20 years, (iii) pregnancy, (iv) uncertain diagnosis of COVID-19, and (v) non-available serum 25(OH)D levels. The cohort included non-obstetric patients aged >20 years who were admitted to our hospital from the emergency department with symptoms consistent with COVID-19, including cough, dyspnea, fever, and/or anosmia from August 1 to October 31, 2020. All participants signed an informed consent form. We engaged in a comparative analysis of 71 patients with 25(OH)D levels available within 48 hours of the admission, with and without vitamin D deficiency (≤20 or >20 ng/ml). The analysis was repeated for the following subgroups: age ≥60 years, male gender, and any comorbidity.

Diagnosis and treatment

Patients were tested for severe acute respiratory syndrome coronavirus-2 (SARS-CoV-2) infection as per local guidelines. For the diagnosis of SARS-CoV-2, RNA was isolated from the nasopharyngeal swab by performing a reverse transcriptase-polymerase chain reaction (RT-PCR) test. The criteria for hospital admission were a combination of oxygen saturation ≤93% at rest, the existence of comorbidities, and the patient's general health status.

All patients with COVID-19 received the available antiviral drugs, empirically validated antibiotics, anticoagulants, corticosteroids, and the standard treatments for comorbidities (Table 1). Chest radiography and thoracic CT scanning were routinely performed in the majority of hospitalized patients.

**Table 1:** Initial dosing strategy for antiviral, low-molecular-weight heparin, and dexamethasone drugs *Dosage depending on body weight and GFR GFR: glomerular filtration rate

Drug	Dosage
Remdesivir (Veklury)	200 mg on day one via intravenous infusion, followed by 100 mg once daily for five days
Azithromycin (Zithromax)	500 mg on day one (oral administration), followed by 250 mg once daily for five days (mild symptoms) or 500 mg once daily for five days (severe symptoms)
Anticoagulants*	
Enoxaparin (Clexane)	Doses of 1 mg/kg (subcutaneous administration) twice daily or 1.5 mg/kg once daily during hospitalization
Tinzaparin (Innohep)	A prophylactic dose of >4,500 IU but <1,75I U/kg (subcutaneous administration) once daily during hospitalization
Dexamethasone phosphate (Dexaton)	8 mg daily via intravenous infusion during hospitalization (severe symptoms)

Data collection

All the data were extracted from the electronic database at the hospital and included demographics (age, gender, comorbidities), the duration of hospitalization, basic laboratory tests, and biochemical markers of infection severity [c-reactive protein (CRP), high-sensitivity troponin I, ferritin, procalcitonin (PCT), D-dimers (DD)], changes in chest radiography, and clinical outcomes (improvement, admission to ICU, mortality).

Outcomes of interest

The main point of interest was 25(OH)D levels, which were assessed within 48 hours of the hospital admission. Serum 25(OH)D levels less than 20 ng/ml were considered to be vitamin D deficiency. The choice of this cut-off value was based on tests in healthy adults, where the administration of vitamin D supplements resulted in a decrease in parathyroid hormone (PTH) in those whose 25(OH)D levels were <20 ng/ml, but not in those whose 25(OH)D levels were >20 ng/ml [2,14].

The primary outcomes of the infection were classified as recovery or mortality during hospitalization. Moreover, admission to ICU and duration of hospital stay were recorded. The secondary outcomes were the maximum values of the inflammatory markers: CRP, ferritin, high-sensitivity troponin I, PCT, and DD. These inflammatory markers were routinely measured during admission and the peak value for each of them was evaluated.

Statistical analysis

The SPSS Statistics software version 26.0 (IBM, Armonk, NY) was used to analyze the collected data. Continuous variables (age, CRP, ferritin, PCT, troponin, DD) are expressed as means and standard deviation while categorical variables (gender, comorbidities, X-ray changes, and mortality) are presented as counts and percentages. The Pearson’s chi-squared test was used for the evaluation of the association between vitamin D deficiency and categorical variables; the Mann-Whitney U test was used to evaluate the association between vitamin D deficiency and continuous variables due to the non-normal distribution of these variables. The level of significance was set at p<0.05.

## Results

A total of 71 patients [mean age: 63 ±17.8 years, range: 20-97; male (n=47; 66.2%); female (n=24; 33.8%)] were included in the study. Among the COVID-19-positive patients, 46 (64.8%) patients had 25(OH)D levels ≤20 ng/ml and 25 (35.2%) patients had a level >20 ng/ml. According to the patients' medical history, 55 (77.5%) of the COVID-19-positive patients had at least one comorbidity (hypertension, diabetes mellitus, cardiovascular disease, and chronic renal disease). Patients with vitamin D deficiency did not differ from those without (vitamin D >20 ng/ml) in terms of age, gender, or comorbidities (Table 2).

**Table 2:** Characteristics of COVID-19 patients classified by serum vitamin D concentrations COVID-19: coronavirus disease 2019; SD: standard deviation; CRD: chronic renal disease; CVD: cardiovascular disease; AD: autoimmune disorder

Characteristics	Total (n=71)	Vitamin D ≤20 ng/ml (n=46)	Vitamin D >20 ng/ml (n=25)	P-value
Age, years, mean ±SD	63 ±17.88	64 ±18.76	62 ±13.95	0.664
Age group, n (%)				0.915
≥60 years	42 (59)	27 (59)	15 (60)	
<60 years	29 (41)	19 (41)	10 (40)	
Gender, n (%)				0.533
Male	47 (66.2)	31 (67.4)	16 (64)	
Female	24 (33.8)	15 (32.6)	9 (36)	
Comorbidities, n (%)				
All	55 (77.5)	35 (76.1)	20 (80.0)	0.706
Hypertension	41 (58)	29 (63)	12 (48)	0.492
Diabetes	17 (24)	10 (22)	7 (28)	0.662
Dementia	9 (12.7)	6 (13)	3 (12)	0.900
CRD	6 (8.5)	5 (10.9)	1 (4)	0.320
Asthma	5 (7)	4 (8.7)	1 (4)	0.900
CVD	18 (25.4)	13 (28.3)	5 (20)	0.117
Hypothyroidism	9 (12.7)	4 (8.7)	5 (20)	0.066
Hyperlipidemia	10 (14.1)	5 (10.9)	5 (20)	0.881
Depression	7 (10)	3 (7)	4 (16)	0.201
AD	6 (8.5)	5 (10.9)	1 (4)	0.656

None of the patients were admitted to the ICU, while the mortality rate was 10.8% in the vitamin D-deficient group and 8% in the normal vitamin D group. The average length of hospital stay was 22 and 13.7 days in the vitamin D-deficient group and the normal vitamin D group, respectively. Patients with vitamin D deficiency had a higher mean CRP (7.94 mg/L vs. 6.67 mg/L), ferritin (298.06 ng/ml vs. 166.07 ng/ml), PCT (0.33 ng/ml vs. 0.26 ng/ml), DD (3,243.02 ng/ml vs. 2,415.6 ng/ml), troponin (44.9 pg/ml vs. 13.25 pg/ml), and increased incidences of radiographic X-ray changes (78.3% vs. 16%) compared to patients with normal vitamin D levels (Table 3).

**Table 3:** Primary and secondary outcome measures classified by serum vitamin D concentrations SD: standard deviation; CRP: c-reactive protein; PCT: procalcitonin; DD: D-dimers

Variables	Vitamin D ≤20 ng/ml (n=46)	Vitamin D >20 ng/ml (n=25)	P-value
CRP, mg/L, mean ±SD	7.94 ±9.11	6.67 ±4.1	0.542
Ferritin, ng/ml, mean ±SD	298.06 ±302.72	166.07 ±95	0.087
PCT, ng/ml, mean ±SD	0.33 ±0.34	0.26 ±0.2	<0.001
DD, ng/ml, mean ±SD	3,243.02 ±4,132	2,415.6 ±3,352.41	0.334
Troponin, pg/ml, mean ±SD	44.9 ±11.28	13.3 ±7.02	<0.001
Length of stay, days, mean ±SD	22 ±11.49	13.7 ±10.52	0.163
X-ray changes, n (%)	36 (78.3)	16 (64)	0.382
Mortality, n (%)	5 (10.9)	2 (8.0)	0.145

## Discussion

The present study demonstrated that vitamin D status, as measured by 25(OH)D concentrations, was not associated with the severity of COVID-19 infection in hospitalized patients. Of the total 71 patients, 64.8% of cases showed a vitamin D deficiency with a longer duration of hospitalization, more radiographic findings, and higher inflammation markers. Also, inflammatory markers (PCT) and cellular damage markers such as troponin were studied, which demonstrated statistically significant differences between the two groups (p<0.001). This suggests that vitamin D deficiency may alter the levels of these biomarkers and may increase the severity of the disease and consequently the risk of death. However, a recently published report did not show differences in troponin and PCT levels between patients who recovered and those who deceased [15].

There is an ongoing debate on whether there is an association between vitamin D deficiency and the severity of COVID-19 infection [16-18]. Baktash et al. have reported no difference in mortality rates between hospitalized COVID-19-positive patients with admission 25(OH)D levels ≤12 ng/ml and those with admission 25(OH)D levels >12 ng/ml. Nevertheless, patients with admission 25(OH)D levels ≤12 ng/ml had increased frequency of admission in ICUs [6]. Additionally, Jevalikar et al. concluded that there was a lack of association of vitamin D status with severity and mortality among Indian patients who were hospitalized for COVID-19 [17]. On the contrary, two studies have found that low concentrations of serum 25(OH)D are significantly associated with a greater extent of lung involvement and poorer outcomes in patients with COVID-19 [7,18]. Similar to previous studies, a retrospective study from Turkey, which analyzed the data of 867 COVID-19 patients, has shown that vitamin D treatment shortened the length of hospitalization and decreased the mortality rate by 2.14 times [19]. Raharusuna et al. have found substantially higher mortality to be correlated with admission 25(OH)D levels <20 ng/ml in 780 hospitalized COVID-19-positive patients in Indonesia [20]. In this study, the overall mortality rate (48.7%) was significantly higher compared with that in our study sample (9.8%). Furthermore, the bigger sample size of the study by Raharusuna et al. enabled the detection of the correlation between vitamin D deficiency and severity of clinical outcomes in COVID-19 infection [20].

Moreover, it should be highlighted that the cut-off vitamin D level to determine the deficiency or depletion of vitamin D is a subject of debate. In our study, 64.8% had vitamin D levels ≤20 ng/ml, which is consistent with prevalence estimates reported by adults in Greece [21]. In our study, vitamin D deficiency was defined as serum levels ≤20 ng/ml, which is sufficient to meet the needs of 97.5% of the general population [22]. We found that age ≥60 years was a risk factor for the severity and poor clinical outcomes of COVID-19 infection. These findings are consistent with the results of previous studies. In two studies from the USA, for every five years of increase in the age of the patient, the hospitalization and mortality rate increased by 34% and 10-18% respectively [23,24].

In our cross-sectional study, there was no significant association between gender and the severity of the clinical outcomes in COVID-19 infection. This finding is consistent with another study by Vasheghani et al. where there was no statistically significant association between male gender and severity and clinical outcomes of COVID-19 infection [25]. On the contrary, in a review article based on findings from three countries (France, Spain, and Switzerland), there was a significant association between the male gender and the severity of COVID-19 inflammation [26]. This could be attributed to hormones in the male gender, concomitant diseases, variations in behavioral characteristics, and greater exposure of men to pathogens. In general, the female gender has a higher immune response against different pathogens due to the protective role of estrogen, facilitating protection against many viral infections [26].

In our study, there was no significant correlation between comorbidities and the severity of COVID-19 infection. This finding is consistent with that of Vasheghani et al., who reported that there was no clear relationship between comorbidities and the severity of COVID-19 infection [25]. Moreover, Bajgain et al. found that there was no association between comorbidities and mortality rate in COVID-19 [27]. This disparity could be due to the different study methods and demographic characteristics of the patients (age and gender) in various studies. On the other hand, there are studies with results that support a significant association between comorbidities and the severity of COVID-19 infection [28,29].

Our study has some limitations. Firstly, the measurement of vitamin D level was performed only once; if the measurement had been carried out several times during the course of the disease, we probably would have had the opportunity to give clear answers with regard to its correlation with the severity and clinical outcomes of COVID-19 infection. Secondly, as our study was a retrospective single-center study, our findings cannot be generalized to other, wider contexts. Furthermore, our data came from Northern Greece, where the population demographics differ from those elsewhere. Third, the number of patients with available vitamin D levels was low, and this may have limited our ability to detect differences between those with and without vitamin D deficiency. Hence our findings must be validated via studies involving larger patient groups, taking into account additional factors such as obesity (as measured by BMI) or other specific comorbidities. Fourth, the measurement of vitamin D levels was performed during August, September, and October. Vitamin D levels are higher during the summer and autumn than in winter and spring [30]. Hence, the timing of the study may have affected the reliability of our findings.

## Conclusions

Based on our findings, there was no association between vitamin D deficiency and the severity and mortality rates of COVID-19 infection. These findings reinforce the need for further multicenter and multinational studies as well as randomized controlled trials in order to clarify the issue and gain deeper insights into it, which would improve the management of patients with COVID-19 infection.
